# Projection-Based Density Matrix Renormalization Group in Density Functional Theory Embedding

**DOI:** 10.1021/acs.jpclett.2c03298

**Published:** 2023-01-17

**Authors:** Pavel Beran, Katarzyna Pernal, Fabijan Pavošević, Libor Veis

**Affiliations:** † J. Heyrovský Institute of Physical Chemistry, Academy of Sciences of the Czech Republic, v.v.i., Dolejškova 3, 18223Prague 8, Czech Republic; ‡ Faculty of Mathematics and Physics, Charles University, 121 16Prague, Czech Republic; ¶ Institute of Physics, Lodz University of Technology, ul. Wolczanska 217/221, 93-005Lodz, Poland; § Center for Computational Quantum Physics, Flatiron Institute, 162 Fifth Avenue, New York, 10010New York, United States

## Abstract

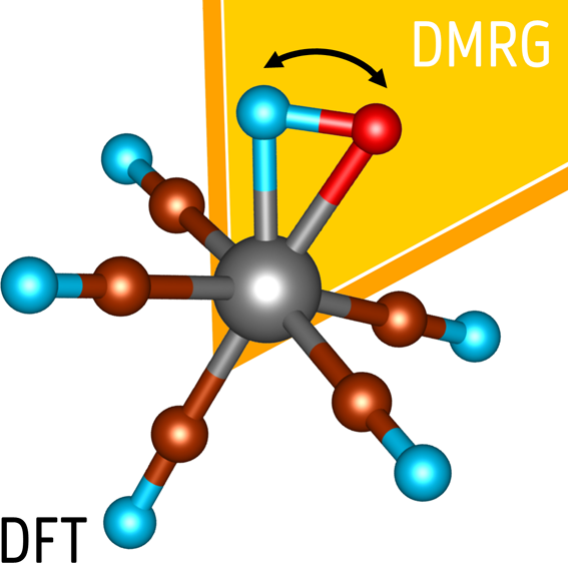

The density matrix renormalization group (DMRG) method has already proved itself as a very efficient and accurate computational method, which can treat large active spaces and capture the major part of strong correlation. Its application on larger molecules is, however, limited by its own computational scaling as well as demands of methods for treatment of the missing dynamical electron correlation. In this work, we present the first step in the direction of combining DMRG with density functional theory (DFT), one of the most employed quantum chemical methods with favorable scaling, by means of the projection-based wave function (WF)-in-DFT embedding. On two proof-of-concept but important molecular examples, we demonstrate that the developed DMRG-in-DFT approach provides a very accurate description of molecules with a strongly correlated fragment.

Strong correlation plays a crucial role in many aspects of chemistry, such as bond-breaking processes, open-shell systems, excited electronic states, as well as in catalysis.^1,2^ Accurate and efficient description of strongly correlated molecules, however, belongs to long-standing challenges of quantum chemistry. In principle, it can be accounted for by the exact full configuration interaction (FCI) method, but it is prohibitively expensive due to its exponential scaling. In order to bypass the limitations of FCI, several approximate polynomially scaling wave function (WF) methods were developed over the years, which can be systematically improved toward FCI. In the case of molecules with weakly correlated electrons, such as organic molecules composed from the main elements and at equilibrium geometries, the most prominent example is undoubtedly the coupled cluster method,^3^ whereas the concept of the complete active space (CAS)^4^ can be considered as a standard tool for strongly correlated molecules, such as transition metal complexes and bond-breaking processes. The last two cases are also the focus of this work.

The complete active space self-consistent field (CASSCF) method,^5^ which couples FCI in a small active space with orbital optimization, is usually the starting point of multireference (MR) calculations. The missing dynamical electron correlation is then taken into account by post-SCF methods, such as the complete active space second-order perturbation theory (CASPT2),^6^ the second-order *n*-electron valence state perturbation theory (NEVPT2),^7^ or the multireference configuration interaction (MRCI).^2^ The common hurdle of all these methods is the limited CAS size of less than 20 orbitals, due to the FCI exponential scaling.

Since many molecules, such as transition metal complexes, require larger CAS than FCI can handle, several approximate FCI solvers have been developed, one of them being the density matrix renormalization group (DMRG) method.^8^ After its introduction in the quantum chemistry,^9^ it has established itself as a powerful technique suitable for generic strongly correlated molecules with a few dozen active orbitals.^10−13^ This sparked interest in development of many post-DMRG methods for treatment of the missing (out-of-CAS) dynamical correlation.^14^ However, these WF-based methods are still too costly for large systems of particular interest. Their alternative, the density functional theory (DFT), represents a cost-effective approach applicable to very large molecules, which, however, has its own limitations. The major shortcomings of DFT are undoubtedly the approximate form of the exchange–correlation functional as well as the single reference character, which makes it unsuitable for strongly correlated problems.^15^

One way of extending the range of applicability of accurate (single or multireference) WF-based methods can be achieved by means of quantum embedding.^16^ This approach relies on locality of chemical interactions and splits the whole system into the active subsystem that is treated at a high level and the environment subsystem that is treated at a lower level of theory.^16,17^ Previously, Neugebauer, Reiher, and co-workers presented the first and to the best of our knowledge the only attempt to embed DMRG calculations in the DFT environment by means of the frozen density embedding approach^18^ for treatment of strongly correlated systems. However, due to the approximate form of the nonadditive kinetic potential (NAKP), their proof-of-principle applications were restricted to systems in which the active subsystem is not covalently bonded to the environment.

The projection-based DFT (PB-DFT) embedding^19^ method is free of the NAKP problem, due to the orthogonality of occupied orbitals of both subsystems, which is achieved by the level shift projection operator.^19^ This additionally ensures that the sum of energies of the active system and the environment effects is equal to the energy of the full system if both fragments are treated at the same level of theory. It is worth mentioning that wave function embedding in DFT computational approaches combines wave function theory with electron density functionals, and generally they may be prone to exchange–correlation double counting. Indeed, in some quantum embedding methods exchange–correlation double counting has been identified as a source of additional errors, and it requires special treatment to improve the overall accuracy.^20^ The frozen density embedding approach^21^ has an exact theoretical foundation and avoids double counting by employing nonadditive kinetic and exchange–correlation functionals. Approximate nonadditive kinetic energy functionals may, however, introduce errors related to kinetic correlation energy double counting in the active system. In the projection-based orbital-embedding, orthogonality of orbitals assigned to different subsystems allows one to avoid exactly double counting of this kind of electron correlation. Alternative embedding schemes free of the double counting of correlation effects comprise, for example, the self-energy embedding theory,^24^ or the subsystem embedding subalgebras^25^ leading to the active space coupled-cluster downfolding techniques.^26^

Encouraged by an impressive performance of the projection-based embedding for various chemical systems such as transition metal catalysis, enzyme reactivity, or battery electrolyte decomposition,^22,23^ as well as by robustness of the DMRG method, herein we develop and implement the DMRG-in-DFT projection-based embedding method. As demonstrated in the remainder of this Letter, this approach has a tremendous potential for applications to large strongly correlated systems.

The DMRG method is a variational procedure for approximating the exact FCI wave function with the so-called matrix product state (MPS).^27^ The FCI wave function in the occupation basis representation reads as
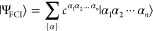
1where occupation of each orbital corresponds to α_*i*_ ∈ {|0⟩, |*↓*⟩, |*↑*⟩, |*↓↑*⟩} and the expansion coefficients  form the FCI tensor. By successive applications of the singular value decomposition (SVD), the FCI tensor can be factorized to the MPS form^27^
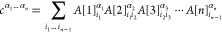
2where  are the MPS matrices specific to each orbital and the newly introduced auxiliary indices *i*_*j*_ are contracted over. If the MPS factorization is exact, the dimensions of the MPS matrices grow in a similar fashion as the size of the original FCI tensor, i.e., exponentially (with an increasing system size). In DMRG, the dimensions of auxiliary indices are bounded. These dimensions are called bond dimensions and are usually denoted with *M*.

A practical version of DMRG is the two-site algorithm, which provides the wave function in the two-site MPS form
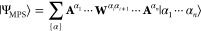
3For a given pair of adjacent indices [*i*, (*i* + 1)], **W** is a four-index tensor, which corresponds to the eigenfunction of the second-quantized electronic Hamiltonian
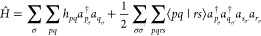
4expanded in the tensor product space of four tensor spaces. The tensor spaces are defined on an ordered orbital chain, so-called left block (*M*_*l*_ dimensional tensor space), left site (four-dimensional tensor space of *i*th orbital), right site (four-dimensional tensor space of (*i* + 1)th orbital), and right block (*M*_*r*_ dimensional tensor space). In eq 4, *h*_*pq*_ and ⟨*pq*|*rs*⟩ denote standard one- and two-electron integrals in the molecular orbital basis, and σ and σ′ denote spin. The MPS matrices **A** are obtained by successive application of SVD with truncation on **W**’s and iterative optimization by going through the ordered orbital chain from left to right and then sweeping back and forth.^11^ The maximum bond dimension (*M*_max_) which is required for a given accuracy can be regarded as a function of the level of entanglement in the studied system.^28^

In the following, we will briefly describe the projection-based embedding WF-in-DFT technique. The WF-in-DFT embedding procedure starts with an initial DFT calculation of the whole system. Based on some criteria for associating the molecular orbitals to the active and environment subsystems, the corresponding density matrix γ is partitioned into the active subsystem A and the environment subsystem B, γ_A_ and γ_B_, respectively. Originally, this was achieved by means of the occupied orbital localization and Mulliken population analysis,^19^ though alternative more robust approaches have also been developed.^29,30^ In the case of the DFT-in-DFT embedded calculation, the total energy can be expressed as^22^
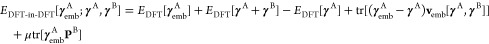
5where *E*_DFT_ denotes the DFT energy evaluated using the bracketed density matrix,  is the embedded subsystem A density matrix, and **P**^B^ is a projection operator enforcing mutual orthogonalization, **P**^B^ = **Sγ**^B^**S**. **S** denotes the atomic orbital overlap matrix. In the limit where the level shift parameter μ → *∞*, the A and B orbitals are exactly orthogonal, but μ is for practical purposes taken to be 10^6^, causing negligible error.^19^ The embedding potential **v**_emb_ contains all interactions between subsystems A and B
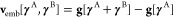
6The matrix **g** groups all the two-electron contributions (Coulomb, exchange, and exchange–correlation). Because, the projection-based embedding approach is free from nonadditive kinetic energy problem^19^ it is formally exact; that is, when the active part was treated with the same exchange–correlation functional as the environment, it would be equivalent to the Kohn–Sham solution of the entire system.

The Fock matrix of subsystem A embedded in B has the following form:^22^
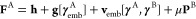
7where **h** is the core Hamiltonian matrix, and it is self-consistently optimized with respect to . In the case of single reference WF-in-DFT calculations, HF-in-DFT with the following effective core Hamiltonian
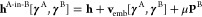
8precedes the WF calculation. For MR problems, CASSCF-in-DFT can be performed.^31^ However, since we employ the accurate DMRG which approaches the FCI solution of the active subsystem, we are free to use HF-in-DFT for the MR problems.

Most importantly, the DFT-in-DFT method can be straightforwardly employed for a WF-in-DFT embedding where the active subsystem is treated with the DMRG method and the environment subsystem is described with the DFT method. Then the DMRG-in-DFT energy is simply obtained by substituting the DFT energy of the active subsystem A with the DMRG energy as
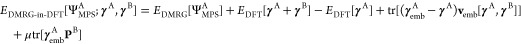
9In this equation,  is the DMRG energy of the active subsystem corresponding to the MPS wave function , which minimizes the active subsystem Hamiltonian (eq 4) with the one-electron part replaced by the effective core Hamiltonian from eq 8.

The WF-in-DFT embedding method has been implemented in Psi4NumPy quantum chemistry software,^32^ which was interfaced with the MOLMPS(33) DMRG code. The developed method was then used to study two benchmark problems (see Figure 1) which have a strongly correlated active part coupled to the environment, namely, the triple bond stretching in propionitrile (CH_3_CH_2_CN) and the conformational isomerization of the model iron-nitrosyl complex [Fe(CN)_5_(NO)]^2–^,^34^ which is a prototype of a transition metal complex with the noninnocent nitrosyl ligand relevant to medicinal applications.^35^ Regarding the low-level method, all the DFT calculations employed the B3LYP,^36,37^ PBE0,^38^ or PBE^39^ density functionals. On the other hand, all the high-level DMRG calculations were warmed-up with the CI-DEAS procedure^11,28^ and took advantage of the dynamical block state selection (DBSS),^40^ which adjusts the actual bond dimensions to fit the desired (preset) truncation error (TRE). The initial DMRG orbital orderings were optimized with the Fiedler method.^41^ The complementary calculations listed below were carried out in the following programs: CCSD in Psi4,^32^ CASSF/DMRG-SCF in Orca,^42^ adiabatic connection (AC) in GammCor,^43^ and internally contracted MRCI in MOLPRO.^44^

**Figure 1:**
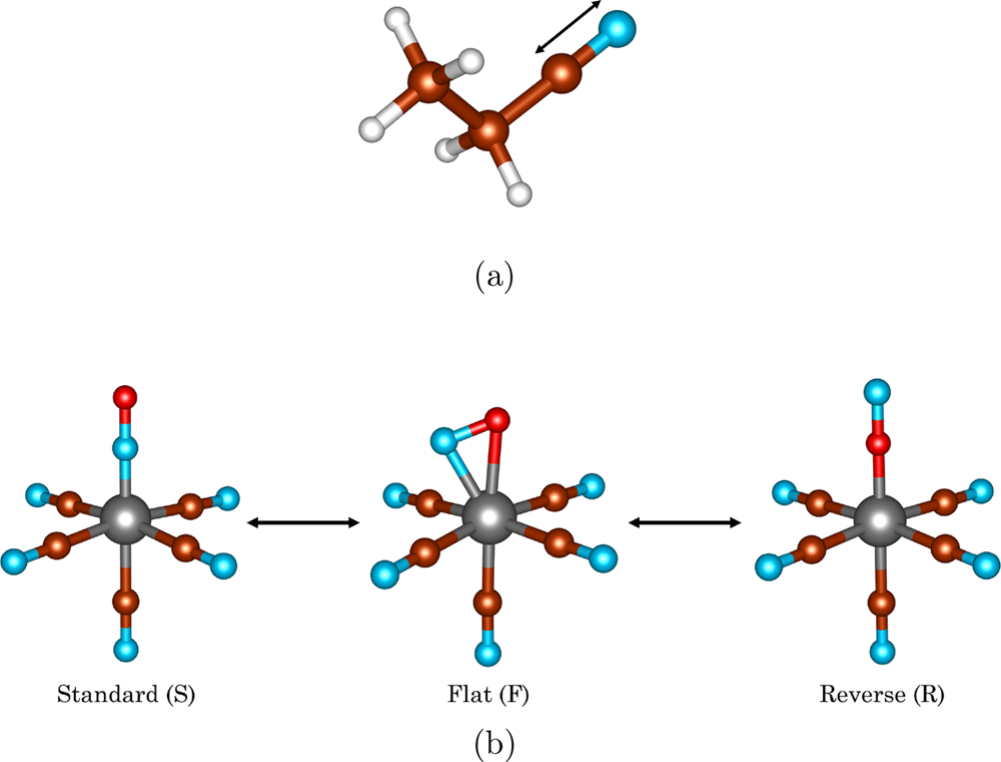
Benchmark problems studied in this work: (a) triple C–N bond stretching in propionitrile (CH_3_CH_2_CN). (b) Conformational isomerization of the [Fe(CN)_5_(NO)]^2–^ complex. The color code is as follows: Fe (gray), N (blue), C (brown), O (red), and H (white).

In our first example, we study the triple bond stretching in propionitrile (CH_3_CH_2_CN) molecule. The equilibrium geometry of propionitrile employed in this work is given in the Supporting Information (Table S1). For the WF-in-DFT calculations, we have employed the cc-pVDZ^45^ basis set. The active subsystem comprised the −CN group, and the orbitals were partitioned into both subsystems by means of the SPADE procedure.^29^ In order to decrease the size of the virtual space, we employed the two-shell concentric localization^46^ leading to the active subsystem FCI space comprising 14 electrons in 63 orbitals. The stretching of the CN bond was probed by the accurate DMRG-in-B3LYP calculations with TRE = 10^–6^. For comparison, we also carried out the CCSD-in-B3LYP, as well as the CCSD and DMRG calculations for the entire molecule. The frozen-core approximation was employed for the aforementioned DMRG calculations leading to the FCI space of 22 electrons in 77 orbitals, and TRE was preset to 10^–5^.

Figure 2, shows the potential energy surfaces (PES) [differences with respect to minima: *E*(*r*_CN_) – *E*_min_] corresponding to the triple C–N bond stretching in propionitrile. The results obtained by B3LYP, CCSD, CCSD-in-B3LYP, and DMRG-in-B3LYP are compared against the exact curve obtained by the frozen-core DMRG method. DMRG-in-PBE and DMRG-in-PBE0 results, which are essentially the same as DMRG-in-B3LYP, are shown in the Supporting Information (Figure S1). The individual absolute energies are provided in Table S2.

**Figure 2:**
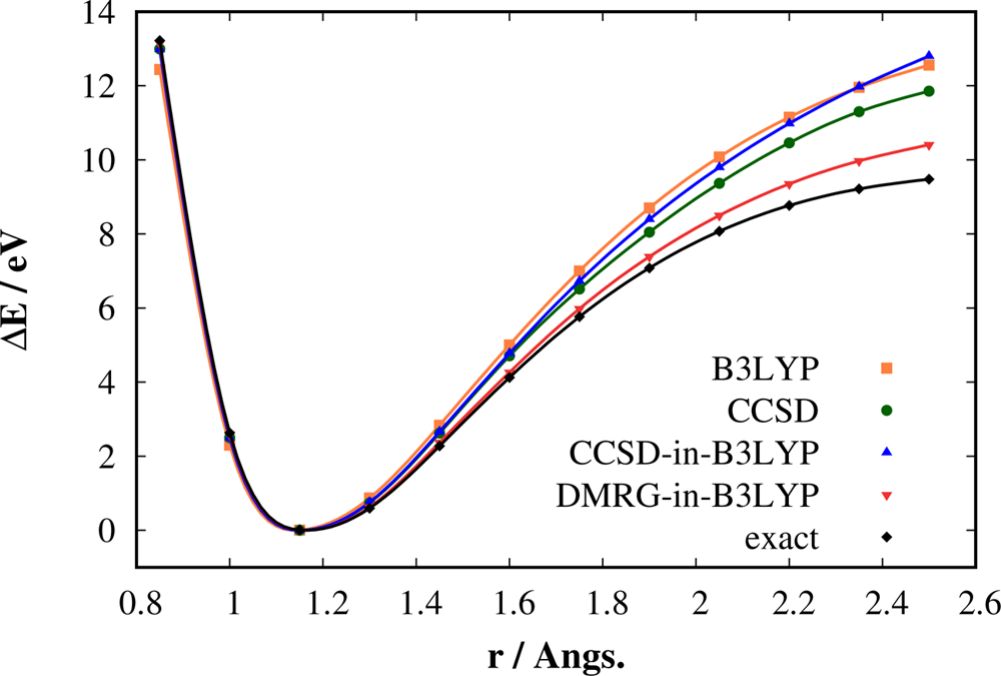
Comparison of the individual dissociation energy curves corresponding to the triple C–N bond stretching in CH_3_CH_2_CN. All calculations employ the cc-pVDZ basis set.

As is well-known, the CCSD method notoriously fails in describing correctly the triple bond breaking due to its single-determinant nature. It, for example, predicts a nonphysical bump on the PES of the N_2_ molecule in the intermediate stretching region (around 2.2 Å).^47^ One can see in Figure 2 that the situation is unsurprisingly very similar for the triple C–N bond stretching in CH_3_CH_2_CN. The CCSD method provides much higher dissociation energies for the intermediate stretching region than the frozen-core DMRG (at 2.5 Å, the error is ∼2.4 eV). CCSD-in-B3LYP behaves even slightly worse than CCSD itself. On the other hand, there is a huge improvement between CCSD-in-B3LYP and DMRG-in-B3LYP in description of the triple C–N bond-stretching process. At 2.5 Å, the error of DMRG-in-B3LYP with respect to DMRG is 0.9 eV, whereas for the CCSD-in-B3LYP method this error is 3.3 eV. The higher-order CC methods would improve the performance of CCSD; nevertheless, they are not suitable for generic multireference problems either. The DMRG method as a genuine MR method is able to properly describe this process. The difference between DMRG-in-B3LYP and DMRG, which is essentially very similar to the difference between CCSD-in-B3LYP and CCSD, thus can be attributed to the lower-level (B3LYP) description of the remaining electrons plus errors of the PB-DFT embedding (density-driven errors or errors of the nonadditive exchange–correlation energy functional^48^). The aforementioned error is not due to the projection since the last term in eqs 5 and 9 is of the order of 10^–9^ a.u. along the whole dissociation curve.

As our second example, we have studied the conformational isomerization of the model iron-nitrosyl complex [Fe(CN)_5_(NO)]^2–^. The B3LYP optimized geometries of the standard, flat, and reversed isomers of [Fe(CN)_5_(NO)]^2–^ (see Figure 1b) were taken from ref (34) (also given in Tables S3–S5). For computational reasons, we used the smaller 6-31G^49,50^ basis. The active subsystem was formed by [Fe–NO]^3+^, and partitioning of the orbitals into subsystems was carried out by means of the SPADE procedure.^29^ We employed the two-shell concentric localization^46^ leading to the active subsystem FCI space comprising 38 electrons in 102 orbitals. For comparison, we also carried out the B3LYP and CCSD calculations as well as calculations with different CAS-based MR methods. [We note that the CCSD reaction energies presented in Table 2 correspond to CCSD preceded by HF with DIIS convergence acceleration (default in Orca(42)). We have found somewhat different HF and consequently also the CCSD energies of S and R isomers with GDM convergence acceleration in Q-Chem.^51^ The resulting Δ*E*_S→R_ is slightly lower than Δ*E*_S→F_ (1.78 and 1.88 eV, respectively) but still much larger than Δ*E*_S→R_ provided by the multireference approaches. Both CCSD absolute energies (Orca^42^ and Q-Chem(51)) can be found in the Supporting Information.] The smallest CAS(4,4) comprising the two NO π* orbitals together with the Fe 3d_*xz*_ and 3d_*yz*_ was employed for internally contracted MRCI with singles and doubles (icMRCISD) calculations. The larger CAS(14,15) contained the NO π (two), π* (two), σ, σ*, and Fe 3d (five), 4d (3 counterparts to the occupied 3d orbitals: 4d_*xy*_, 4d_*xz*_, and 4d_*yz*_), plus one equatorial σ orbital with the Fe  and C 2p_*x*/*y*_ contributions. This CAS(14,15) was augmented with one occupied axial orbital of σ character to form CAS(16,16). All CASSCF natural orbitals are shown in Figures S2–S10). In the smaller CAS(14,15), we performed CASSCF computations, which were then corrected for the dynamical electron correlation by means of strongly contracted NEVPT2, the adiabatic connection (AC),^52,53^ and the linearized-AC-integrand approximation AC0.^52,53^ The later two have the advantage of favorable scaling with respect to the CAS size and thus represent an ideal choice for approximate FCI solvers such as DMRG.^54^ In CAS(16,16), we performed the DMRG-SCF calculations with fixed bond dimensions equal to 2000 and subsequent AC/AC0 in order to probe the effect of the missing dynamical electron correlation.

Table 1 shows the natural orbital occupation numbers (NOONs) of the four orbitals around the Fermi level for the largest active space employed, i.e., CAS(16,16) (all occupation numbers can be found in the Supporting Information).

**Table 1:** DMRG-SCF(16,16) Natural Orbital Occupation Numbers for the Individual [Fe(CN)_5_(NO)]^2–^ Standard (S), Flat (F), and Reverse (R) Isomers

isomer	HOMO–1	HOMO	LUMO	LUMO+1
S	1.82	1.82	0.21	0.21
F	1.92	1.77	0.25	0.10
R	1.72	1.72	0.32	0.32

The occupation numbers largely deviate from 2 (and 0) and confirm the noninnocent nature of the nitrosyl ligand, indicating the significant multireference character of the investigated systems. Moreover, looking at the four aforementioned orbitals (Figures S8–S10), one can see that their electron density is mainly localized to the Fe–NO region, which corroborates the use of the WF-in-DFT embedding, in which the WF method, however, should be able to correctly describe the MR character of the Fe–NO moiety. The strongest MR character is observed for the reverse isomer. In this case, the weight of the HF reference in the DMRG-SCF(16,16) wave function is only 64%, and one can expect that the conventional single-reference approaches might be inappropriate.

Table 2 shows the reaction energies of three stable isomers involved in the [Fe(CN)_5_(NO)]^2–^ complex conformational isomerization computed by various single and multireference methods as well as with the CCSD and DMRG methods embedded in the HF or DFT environment. The graphical summary is depicted in Figure 3. Because of the significant multireference character in all three isomers, Figure 3 and Table 2 indicate that the single-reference methods (B3LYP and CCSD), in contrast to all state-of-the-art multireference approaches, incorrectly predict the reverse isomer to have the highest energy. At the CAS(14,15) level, we can observe that adding the dynamical electron correlation on top of CASSCF by means of NEVPT2 and AC0/AC results in a larger Δ*E*_S→F_ by 0.6–0.7 eV, whereas Δ*E*_S→R_ is affected only slightly. More importantly, AC0 provides very similar energy gaps as NEVPT2 (within 0.16 eV in the case of Δ*E*_S→R_), as was already pointed out previously.^53^ The canonical AC method captures even more correlation energy than its linearized AC0 approximation, and the AC(16,16) results together with the icMRCISD(4,4) results represent our best estimates of the energy gaps, in particular 1.9–2.14 eV for Δ*E*_S→F_ and ∼1.40 eV for Δ*E*_S→R_.

**Table 2:** Reaction Energies (in eV) Corresponding to the Conformational Isomerization of [Fe(CN)_5_(NO)]^2–^ Complex Calculated with Different Methods and 6-31G Basis Set

	Δ*E*_S→F_^a^	Δ*E*_S→R_^b^
B3LYP	1.77	1.91
CCSD	1.72	2.03
CASSCF(14,15)	1.63	1.23
NEVPT2(14,15)	2.30	1.34
AC0(14,15)	2.34	1.18
AC(14,15)	2.20	1.15
DMRG-SCF(16,16)	1.83	1.18
AC0(16,16)	2.18	1.46
AC(16,16)	2.14	1.38
icMRCISD(4,4)	1.90	1.44
CCSD-in-B3LYP	1.27	1.85
CCSD-in-HF	1.36	2.12
DMRG-in-B3LYP	1.92	1.17
DMRG-in-PBE0	1.89	1.27
DMRG-in-HF	2.01	1.44

a Δ*E*_S→F_ denotes the energy difference between flat (F) and standard (S) isomers.

b Δ*E*_S→R_ denotes the energy difference between reverse (R) and standard (S) isomers.

**Figure 3:**
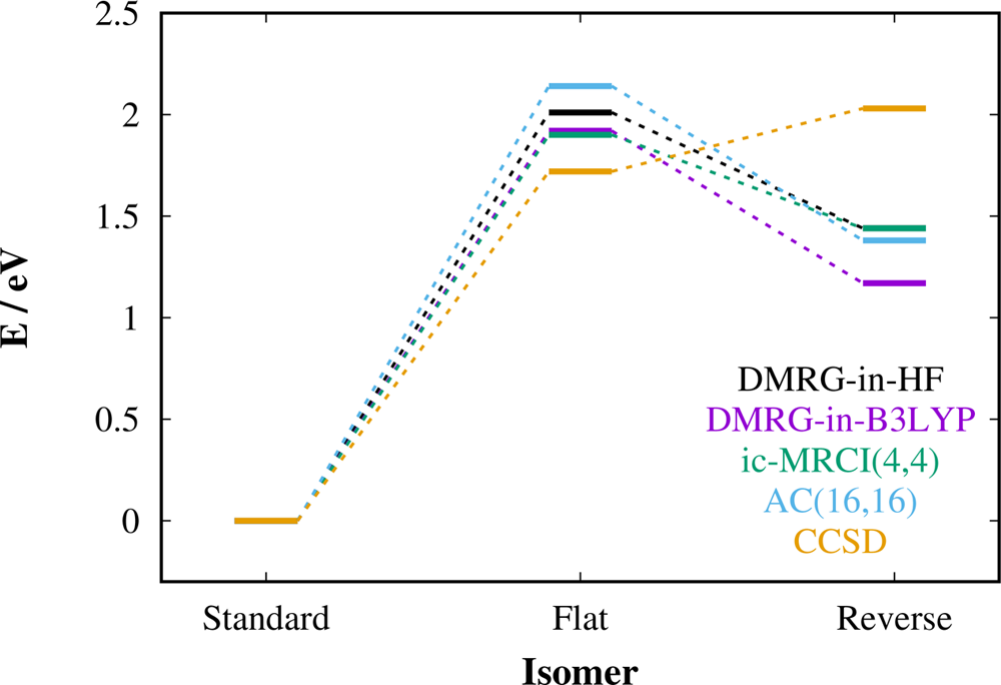
Graphical representation of energetics of [Fe(CN)_5_(NO)]^2–^ complex conformational isomerization for selected computational methods.

Looking at the results of the embedded calculations in Table 2, one can see that CCSD-in-HF as well as CCSD-in-B3LYP underestimate the Δ*E*_S→F_ gap even more than CCSD and predict incorrectly that the flat isomer is lower in energy than the reverse one (by 0.8 and 0.6 eV, respectively). In contrast, the results of the DMRG embedded calculations are in a very good agreement with our best estimates of the energy gaps. Both DMRG-in-HF as well as DMRG-in-B3LYP provide Δ*E*_S→F_ gaps within the margins of the MR methods, and the DMRG-in-B3LYP Δ*E*_S→R_ gap is slightly lower (by ∼0.2 eV). The difference between DMRG-in-B3LYP and DMRG-in-PBE0 is fractionally higher than in the case of propionitrile (0.03 and 0.1 eV for Δ*E*_S→F_ and Δ*E*_S→R_, respectively) and may point to larger errors of nonadditive exchange–correlation energy functional in transition metal complexes.^55^ The DMRG-in-HF method achieves a perfect agreement of both energy gaps with our best estimates obtained by the state-of-the-art MR methods, which confirms that the Fe-NO moiety is mainly responsible for the electronic structure properties of the [Fe(CN)_5_(NO)]^2–^ complex.

In this Letter, we present the projection-based DMRG-in-DFT embedding method and we test its performance on two benchmark problems, namely, the triple bond stretching in CH_3_CH_2_CN and conformational isomerization of [Fe(CN)_5_(NO)]^2–^, a prototype of the transition metal complex containing a noninnocent ligand. Both of these systems exhibit a significant multireference character. Our numerical results indicate that the DMRG-in-DFT provides a viable way toward accurate description of molecules containing strongly correlated fragment. In the case of the triple bond stretching in CH_3_CH_2_CN, the DMRG-in-B3LYP method substantially outperformed the single-reference CCSD and CCSD-in-B3LYP methods, whereas in the case of the [Fe(CN)_5_(NO)]^2–^ complex, the DMRG-in-B3LYP and DMRG-in-HF methods provided the energy gaps between individual isomers that are in very good agreement with the state-of-the-art multireference approaches. This work represents the first step toward combining DMRG with PB-DFT embedding. The biggest bottleneck of this approach is the size of the virtual space which, even when it is truncated,^46^ might be too large for DMRG. It is also the reason why we were limited to smaller basis sets. However, in the case of larger basis sets, the concept of CAS can be used in which DMRG is combined with some post-DMRG method.^14^ Particularly appealing is a connection of DMRG-SCF-in-DFT with the AC methodology^52,53,56^ due to its favorable scaling with the CAS size and quick convergence with the DMRG bond dimensions.^54^ This combination will be the subject of our following works.

## Supporting Information Available

Equilibrium geometry of CH_3_CH_2_CN; all computed absolute energies of CH_3_CH_2_CN for the C–N bond stretching; comparison of DMRG-in-DFT with different DFT functionals for CH_3_CH_2_CN; geometries of standard, flat, and reversed isomers of [Fe(CN)_5_(NO)]^2–^; all computed absolute energies of standard, flat, and reversed isomers of [Fe(CN)_5_(NO)]^2–^; CASSCF and DMRG-SCF natural orbitals and occupation numbers of [Fe(CN)_5_(NO)]^2–^ (PDF)

## References

[ref1] Lyakh D. I.; Musiał M.; Lotrich V. F.; Bartlett R. J. Multireference Nature of Chemistry: The Coupled-cluster View. Chem. Rev. 2012, 112, 182–243. 10.1021/cr2001417. 22220988

[ref2] Szalay P. G.; Müller T.; Gidofalvi G.; Lischka H.; Shepard R. Multiconfiguration Self-Consistent Field and Multireference Configuration Interaction Methods and Applications. Chem. Rev. 2012, 112, 108–181. 10.1021/cr200137a. 22204633

[ref3] Bartlett R. J.; Musiał M. Coupled-cluster Theory in Quantum Chemistry. Rev. Mod. Phys. 2007, 79, 291–352. 10.1103/RevModPhys.79.291.

[ref4] Roos B. O. The Complete Active Space Self-Consistent Field Method and its Applications in Electronic Structure Calculations. Adv. Chem. Phys. 2007, 69, 399–445. 10.1002/9780470142943.ch7.

[ref5] Roos B. O.; Taylor P. R.; Sigbahn P. E. A Complete Active Space SCF Method (CASSCF) using a Density Matrix Formulated Super-CI Approach. Chem. Phys. 1980, 48, 157–173. 10.1016/0301-0104(80)80045-0.

[ref6] Andersson K.; Malmqvist P.-Å.; Roos B. O. Second-order Perturbation Theory with a Complete Active Space Self-consistent Field Reference Function. J. Chem. Phys. 1992, 96, 1218–1226. 10.1063/1.462209.

[ref7] Angeli C.; Cimiraglia R.; Evangelisti S.; Leininger T.; Malrieu J.-P. Introduction of n-electron Valence States for Multireference Perturbation Theory. J. Chem. Phys. 2001, 114, 10252–10264. 10.1063/1.1361246.

[ref8] White S. R. Density Matrix Formulation for Quantum Renormalization Groups. Phys. Rev. Lett. 1992, 69, 2863 10.1103/PhysRevLett.69.2863. 10046608

[ref9] White S. R.; Martin R. L. Ab Initio Quantum Chemistry using the Density Matrix Renormalization Group. J. Chem. Phys. 1999, 110, 4127–4130. 10.1063/1.478295.

[ref10] Chan G. K.-L.; Sharma S. The Density Matrix Renormalization Group in Quantum Chemistry. Annu. Rev. Phys. Chem. 2011, 62, 465–481. 10.1146/annurev-physchem-032210-103338. 21219144

[ref11] Szalay S.; Pfeffer M.; Murg V.; Barcza G.; Verstraete F.; Schneider R.; Legeza Ö. Tensor Product Methods and Entanglement Optimization for Ab Initio Quantum Chemistry. Int. J. Quantum Chem. 2015, 115, 1342–1391. 10.1002/qua.24898.

[ref12] Yanai T.; Kurashige Y.; Mizukami W.; Chalupskỳ J.; Lan T. N.; Saitow M. Density Matrix Renormalization Group for Ab Initio Calculations and Associated Dynamic Correlation Methods: A Review of Theory and Applications. Int. J. Quantum Chem. 2015, 115, 283–299. 10.1002/qua.24808.

[ref13] Baiardi A.; Reiher M. The Density Matrix Renormalization Group in Chemistry and Molecular Physics: Recent Developments and new Challenges. J. Chem. Phys. 2020, 152, 040903 10.1063/1.5129672. 32007028

[ref14] Cheng Y.; Xie Z.; Ma H. Post-Density Matrix Renormalization Group Methods for Describing Dynamic Electron Correlation with Large Active Spaces. J. Phys. Chem. Lett. 2022, 13, 904–915. 10.1021/acs.jpclett.1c04078. 35049302

[ref15] Burke K. Perspective on Density Functional Theory. J. Chem. Phys. 2012, 136, 150901 10.1063/1.4704546. 22519306

[ref16] Jones L. O.; Mosquera M. A.; Schatz G. C.; Ratner M. A. Embedding Methods for Quantum Chemistry: Applications from Materials to Life Sciences. J. Am. Chem. Soc. 2020, 142, 3281–3295. 10.1021/jacs.9b10780. 31986877

[ref17] Sun Q.; Chan G. K.-L. Quantum Embedding Theories. Acc. Chem. Res. 2016, 49, 2705–2712. 10.1021/acs.accounts.6b00356. 27993005

[ref18] Dresselhaus T.; Neugebauer J.; Knecht S.; Keller S.; Ma Y.; Reiher M. Self-consistent Embedding of Density-matrix Renormalization Group Wavefunctions in a Density Functional Environment. J. Chem. Phys. 2015, 142, 044111 10.1063/1.4906152. 25637973

[ref19] Manby F. R.; Stella M.; Goodpaster J. D.; Miller T. F. A Simple, Exact Density-Functional-Theory Embedding Scheme. J. Chem. Theory Comput.h 2012, 8, 2564–2568. 10.1021/ct300544e. PMC3419460 22904692

[ref20] Ma H.; Sheng N.; Govoni M.; Galli G. Quantum embedding theory for strongly correlated states in materials. J. Chem. Theory Comput. 2021, 17, 2116–2125. 10.1021/acs.jctc.0c01258. 33739106

[ref21] Wesolowski T. A.; Shedge S.; Zhou X. Frozen-density embedding strategy for multilevel simulations of electronic structure. Chem. Rev. 2015, 115, 5891–5928. 10.1021/cr500502v. 25923542

[ref24] Lan T. N.; Kananenka A. A.; Zgid D. Communication: Towards ab initio self-energy embedding theory in quantum chemistry. J. Chem. Phys. 2015, 143, 241102 10.1063/1.4938562. 26723581

[ref25] Kowalski K. Properties of coupled-cluster equations originating in excitation sub-algebras. J. Chem. Phys. 2018, 148, 094104 10.1063/1.5010693.

[ref26] Bauman N. P.; Kowalski K. Coupled cluster downfolding methods: The effect of double commutator terms on the accuracy of ground-state energies. J. Chem. Phys. 2022, 156, 094106 10.1063/5.0076260. 35259890

[ref22] Lee S. J. R.; Welborn M.; Manby F. R.; Miller T. F. Projection-Based Wavefunction-in-DFT Embedding. Acc. Chem. Res. 2019, 52, 1359–1368. 10.1021/acs.accounts.8b00672. 30969117

[ref23] Pavošević F.; Rubio A. Wavefunction Embedding for Molecular Polaritons. J. Chem. Phys. 2022, 157, 094101 10.1063/5.0095552. 36075718

[ref27] Schollwöck U. The Density-matrix Renormalization Group in the Age of Matrix Product States. Ann. Phys. 2011, 326, 96–192. 10.1016/j.aop.2010.09.012.

[ref28] Legeza O.; Sólyom J. Optimizing the Density-matrix Renormalization Group Method using Quantum Information Entropy. Phys. Rev. B 2003, 68, 195116 10.1103/PhysRevB.68.195116.

[ref29] Claudino D.; Mayhall N. J. Automatic Partition of Orbital Spaces Based on Singular Value Decomposition in the Context of Embedding Theories. J. Chem. Theory Comput.h 2019, 15, 1053–1064. 10.1021/acs.jctc.8b01112. 30620604

[ref30] Waldrop J. M.; Windus T. L.; Govind N. Projector-Based Quantum Embedding for Molecular Systems: An Investigation of Three Partitioning Approaches. J. Phys. Chem. A 2021, 125, 6384–6393. 10.1021/acs.jpca.1c03821. 34260852

[ref31] de Lima Batista A. P.; de Oliveira-Filho A. G. S.; Galembeck S. E. Photophysical Properties and the NO Photorelease Mechanism of a Ruthenium Nitrosyl Model Complex Investigated using the CASSCF-in-DFT Embedding Approach. Phys. Chem. Chem. Phys. 2017, 19, 13860–13867. 10.1039/C7CP01642E. 28513675

[ref32] Smith D. G. A.; et al. Psi4NumPy: An Interactive Quantum Chemistry Programming Environment for Reference Implementations and Rapid Development. J. Chem. Theory Comput.h 2018, 14, 3504–3511. 10.1021/acs.jctc.8b00286. 29771539

[ref33] Brabec J.; Brandejs J.; Kowalski K.; Xantheas S.; Legeza Ö.; Veis L. Massively Parallel Quantum Chemical Density Matrix Renormalization Group Method. J. Comput. Chem. 2021, 42, 534–544. 10.1002/jcc.26476. 33377527

[ref34] Daniel C.; Gourlaouen C. Structural and Optical Properties of Metal-Nitrosyl Complexes. Molecules 2019, 24, 3638 10.3390/molecules24203638. 31600965 PMC6832229

[ref35] Awasabisah D.; Richter-Addo G. In Adv. Inorg. Chem.; Van Eldik R., Olabe J., Eds.; Academic Press: Cambridge, MA, 2015; Vol. 67; Chapter NOx Related Chemistry, pp 1–86.

[ref36] Lee C.; Yang W.; Parr R. G. Development of the Colle-Salvetti Correlation-Energy Formula into a Functional of the Electron Density. Phys. Rev. B 1988, 37, 785–789. 10.1103/PhysRevB.37.785. 9944570

[ref37] Becke A. D. Density-Functional Exchange-Energy Approximation with Correct Asymptotic Behavior. Phys. Rev. A 1988, 38, 3098–3100. 10.1103/PhysRevA.38.3098. 9900728

[ref38] Perdew J. P.; Ernzerhof M.; Burke K. Rationale for mixing exact exchange with density functional approximations. J. Chem. Phys. 1996, 105, 9982–9985. 10.1063/1.472933.

[ref39] Perdew J. P.; Burke K.; Ernzerhof M. Generalized Gradient Approximation Made Simple. Phys. Rev. Lett. 1996, 77, 3865–3868. 10.1103/PhysRevLett.77.3865. 10062328

[ref40] Legeza Ö.; Röder J.; Hess B. Controlling the Accuracy of the Density-matrix Renormalization-Group Method: The Dynamical Block State Selection Approach. Phys. Rev. B 2003, 67, 125114 10.1103/PhysRevB.67.125114.

[ref41] Barcza G.; Legeza O.; Marti K. H.; Reiher M. Quantum-information Analysis of Electronic States of Different Molecular Structures. Phys. Rev. A 2011, 83, 012508 10.1103/PhysRevA.83.012508.

[ref42] Neese F. The ORCA Program System. WIREs Comput. Mol. Sci. 2012, 2, 73–78. 10.1002/wcms.81.

[ref43] Pernal K.; Hapka M.; Przybytek M.; Modrzejewski M.; Sokół A. GammCor code. https://github.com/pernalk/GAMMCOR, 2022.

[ref44] Werner H.-J.; et al. The Molpro Quantum Chemistry Package. J. Chem. Phys. 2020, 152, 144107 10.1063/5.0005081. 32295355

[ref45] Dunning T. H. Gaussian Basis Sets for use in Correlated Molecular Calculations. I. The Atoms Boron Through Neon and Hydrogen. J. Chem. Phys. 1989, 90, 1007–1023. 10.1063/1.456153.

[ref46] Claudino D.; Mayhall N. J. Simple and Efficient Truncation of Virtual Spaces in Embedded Wave Functions via Concentric Localization. J. Chem. Theory Comput.h 2019, 15, 6085–6096. 10.1021/acs.jctc.9b00682. 31545600

[ref47] Kinoshita T.; Hino O.; Bartlett R. J. Coupled-cluster Method Tailored by Configuration Interaction. J. Chem. Phys. 2005, 123, 074106 10.1063/1.2000251. 16229558

[ref48] Goodpaster J. D.; Barnes T. A.; Manby F. R.; Miller T. F. Accurate and Systematically Improvable Density Functional Theory Embedding for Correlated Wavefunctions. J. Chem. Phys. 2014, 140, 18A507 10.1063/1.4864040. 24832315

[ref49] Hehre W. J.; Ditchfield R.; Pople J. A. Self—Consistent Molecular Orbital Methods. XII. Further Extensions of Gaussian—Type Basis Sets for Use in Molecular Orbital Studies of Organic Molecules. J. Chem. Phys. 1972, 56, 2257–2261. 10.1063/1.1677527.

[ref50] Rassolov V. A.; Pople J. A.; Ratner M. A.; Windus T. L. 6-31G* Basis Set for Atoms K Through Zn. J. Chem. Phys. 1998, 109, 1223–1229. 10.1063/1.476673.

[ref51] Epifanovsky E.; Gilbert A. T.; Feng X.; Lee J.; Mao Y.; Mardirossian N.; Pokhilko P.; White A. F.; Coons M. P.; Dempwolff A. L.; et al. Software for the frontiers of quantum chemistry: An overview of developments in the Q-Chem 5 package. J. Chem. Phys. 2021, 155, 084801 10.1063/5.0055522. 34470363 PMC9984241

[ref52] Pernal K. Electron Correlation from the Adiabatic Connection for Multireference Wave Functions. Phys. Rev. Lett. 2018, 120, 013001 10.1103/PhysRevLett.120.013001. 29350961

[ref53] Pastorczak E.; Pernal K. Correlation Energy from the Adiabatic Connection Formalism for Complete Active Space Wave Functions. J. Chem. Theory Comput. 2018, 14, 3493–3503. 10.1021/acs.jctc.8b00213. 29787257

[ref54] Beran P.; Matoušek M.; Hapka M.; Pernal K.; Veis L. Density Matrix Renormalization Group with Dynamical Correlation via Adiabatic Connection. J. Chem. Theory Comput.h 2021, 17, 7575–7585. 10.1021/acs.jctc.1c00896. 34762423

[ref55] Bensberg M.; Neugebauer J. Density functional theory based embedding approaches for transition-metal complexes. Phys. Chem. Chem. Phys. 2020, 22, 26093–26103. 10.1039/D0CP05188H. 33201953

[ref56] Drwal D.; Beran P.; Hapka M.; Modrzejewski M.; Sokół A.; Veis L.; Pernal K. Efficient Adiabatic Connection Approach for Strongly Correlated Systems: Application to Singlet–Triplet Gaps of Biradicals. J. Phys. Chem. Lett. 2022, 13, 4570–4578. 10.1021/acs.jpclett.2c00993. 35580342 PMC9150121

