# Supplementary material for: Projection-Based Density Matrix Renormalization Group in Density Functional Theory Embedding
Source: J Phys Chem Lett. 2023 Jan 17;14(3):716–22. doi: 10.1021/acs.jpclett.2c03298 (PMC10017021; doi:10.1021/acs.jpclett.2c03298)
Supplement: Supplementary file 1 — jz2c03298_si_001.pdf [file jz2c03298_si_001.pdf]

Supporting Information: Projection-based Density Matrix Renormalization Group in Density Functional Theory Embedding

Pavel Beran,[†,‡] Katarzyna Pernal,[¶] Fabijan Pavošević,[*,§] and Libor Veis[*,†]

†*J. Heyrovský Institute of Physical Chemistry, Academy of Sciences of the Czech Republic, v.v.i., Dolejškova 3, 18223 Prague 8, Czech Republic*

‡*Faculty of Mathematics and Physics, Charles University, Prague, Czech Republic*

¶*Institute of Physics, Lodz University of Technology, ul. Wolczanska 217/221, 93-005 Lodz, Poland*

§*Center for Computational Quantum Physics, Flatiron Institute, 162 5th Ave., New York, 10010 NY, USA*

E-mail: fpavosevic@gmail.com; libor.veis@jh-inst.cas.cz

Propionitrile (CH_3CH_2CN) C-N bond stretching

Table S1: Equilibrium geometry of CH_3CH_2CN, XYZ in Å.

C	-2.38207	-0.46087	0.01893
N	-3.18147	-0.80786	0.76930
H	-0.03176	0.93909	0.54131
C	0.02231	0.17867	-0.25262
H	0.75941	0.50795	-1.00054
H	0.38038	-0.75999	0.19640
C	-1.34723	-0.01758	-0.92251
H	-1.69162	0.92161	-1.38724
H	-1.28110	-0.76419	-1.73200

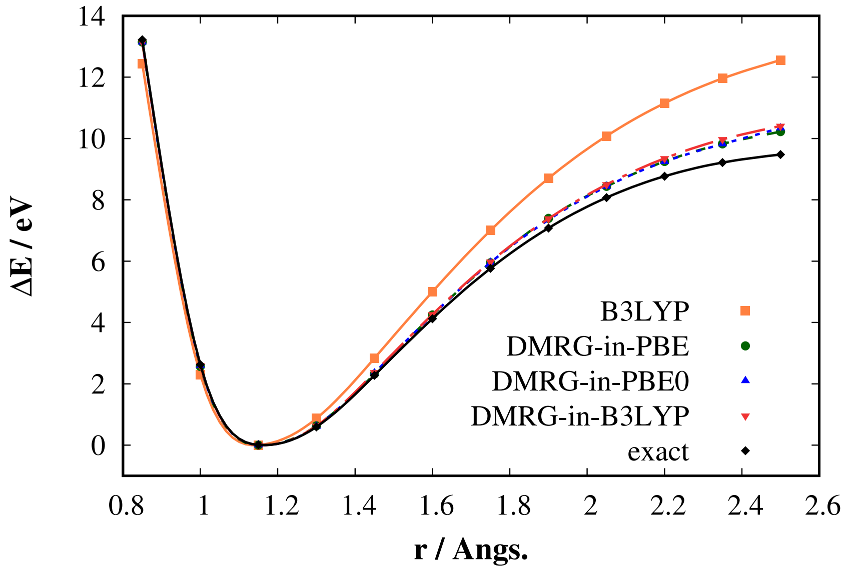

Figure S1: Comparison of DMRG-in-DFT dissociation energy curves with different DFT functionals (B3LYP, PBE0, PBE) corresponding to the triple C-N bond stretching in CH_3CH_2CN. All calculations employ the cc-pVDZ basis set.

Table S2: Absolute energies of CH_3CH_2CN for a given C-N bond length (in Å). All calculations were performed in the cc-pVDZ basis, energies are listed in a.u., and FC denotes the frozen-core approximation. DMRG(FC) calculations were performed with the DBSS procedure and TRE=10^{-5}.

$r_{\text{C-N}}$	B3LYP	CCSD	CCSD-in-B3LYP	DMRG(FC)
0.85	-171.618382	-171.106586	-171.337520	-171.11161047
1.00	-171.991004	-171.492235	-171.723038	-171.50086928
1.15	-172.075359	-171.584162	-171.814725	-171.59716505
1.30	-172.043567	-171.556873	-171.786935	-171.57557639
1.45	-171.971320	-171.487757	-171.717006	-171.51378665
1.60	-171.891661	-171.411147	-171.639154	-171.44582726
1.75	-171.818215	-171.344743	-171.567481	-171.38558935
1.90	-171.755710	-171.288331	-171.506139	-171.33720854
2.05	-171.705096	-171.239879	-171.454591	-171.30076817
2.20	-171.665708	-171.199782	-171.411130	-171.27519061
2.35	-171.636018	-171.168898	-171.374676	-171.25875127
2.50	-171.614037	-171.148550	-171.344320	-171.24910098
$r_{\text{C-N}}$	DMRG-in-B3LYP	DMRG-in-PBE	DMRG-in-PBE0	DMRG(FC)
0.85	-171.341987	-171.21918	-171.24037	-171.11161047
1.00	-171.730008	-171.60788	-171.62800	-171.50086928
1.15	-171.825192	-171.70209	-171.72315	-171.59716505
1.30	-171.802364	-171.67906	-171.70089	-171.57557639
1.45	-171.738241	-171.61693	-171.63709	-171.51378665
1.60	-171.668690	-171.54621	-171.56718	-171.44582726
1.75	-171.605607	-171.48355	-171.50454	-171.38558935
1.90	-171.553929	-171.43037	-171.45251	-171.33720854
2.05	-171.513078	-171.39197	-171.41332	-171.30076817
2.20	-171.481714	-171.36223	-171.38363	-171.27519061
2.35	-171.458972	-171.34112	-171.36242	-171.25875127
2.50	-171.442953	-171.32648	-171.34336	-171.24910098

$[Fe(CN)_5(NO)]^{2-}$ complex conformational isomerization

Geometries

Source: SI of Daniel, C.; Gourlaouen, C. Structural and Optical Properties of Metal-Nitrosyl Complexes. Molecules 2019, 24, 3638.

Table S3: Geometry of the standard isomer of $[Fe(CN)_5(NO)]^{2-}$ complex, XYZ in Å.

Fe	0.00149500	-0.00106700	-0.09336900
C	-0.03222200	0.02103400	1.86410100
C	-1.75416100	0.85771400	0.02002800
C	0.85659200	1.75411800	0.05268600
C	1.75258100	-0.85664900	0.09511900
C	-0.85785400	-1.75326800	0.06284700
N	-0.05222500	0.03386700	3.03384600
N	2.80100800	-1.36744800	0.19103400
N	-1.37158600	-2.80173800	0.14115000
N	1.36881800	2.80371400	0.12573200
N	-2.80414500	1.37165900	0.07265600
N	0.02861100	-0.01928200	-1.73124100
O	0.04726700	-0.03192100	-2.87166600

Table S4: Geometry of the flat isomer of $[Fe(CN)_5(NO)]^{2-}$ complex, XYZ in Å.

Fe	-0.00016900	0.02806300	-0.12906000
C	0.00035300	-0.17615400	1.76755000
C	-1.38040600	1.40923600	0.04432800
C	1.37930400	1.41018200	0.04440300
C	1.48359600	-1.27200700	-0.02254300
C	-1.48287100	-1.27288100	-0.02213100
N	0.00074600	-0.27164900	2.93388700
N	2.36724000	-2.03798600	0.03986000
N	-2.36594400	-2.03946500	0.04094100
N	2.19600000	2.24440400	0.13982500
N	-2.19754400	2.24305200	0.13952600
N	0.00022500	-0.74637000	-1.85226200
O	-0.00006700	0.36702500	-2.20081400

Table S5: Geometry of the reverse isomer of $[Fe(CN)_5(NO)]^{2-}$ complex, XYZ in Å.

Fe	-0.00060500	0.00018400	-0.05436100
C	0.03567000	-0.02895700	1.86383700
C	-1.53095900	1.21983800	0.09791900
C	1.22394800	1.53034400	0.04912800
C	1.53359200	-1.22166100	0.00102400
C	-1.21846800	-1.53465800	0.04870100
N	0.05817700	-0.04746800	3.03450200
N	2.44981100	-1.95160100	0.02040000
N	-1.94702900	-2.45095200	0.09594500
N	1.95614400	2.44363500	0.09789000
N	-2.44506900	1.94860300	0.17478400
O	-0.03787600	0.03041200	-1.80130200
N	-0.06403000	0.05242500	-2.92921600

Energies

Table S6: Absolute energies (in a.u.) of standard, flat, and reverse isomers of $[Fe(CN)_5(NO)]^{2-}$ complex in 6-31G basis.

	standard	flat	reverse
B3LYP	-1857.204433	-1857.139493	-1857.134402
CCSD^a	-1854.298712	-1854.235608	-1854.224112
CCSD^b	-1854.344365	-1854.275324	-1854.278839
CASSCF(14,15)	-1852.95065	-1852.890706	-1852.905416
NEVPT2(14,15)	-1854.268748	-1854.184329	-1854.219546
AC0(14,15)	-1854.294758	-1854.208607	-1854.251568
AC(14,15)	-1854.089513	-1854.008786	-1854.047418
DMRG-SCF(16,16)	-1852.997355	-1852.930239	-1852.953831
AC0(16,16)	-1854.304948	-1854.224917	-1854.251228
AC(16,16)	-1854.107123	-1854.028369	-1854.05652
icMRCISD(4,4)	-1853.756956	-1853.686976	-1853.703916
CCSD-in-B3LYP	-1856.113810	-1856.067165	-1856.045671
CCSD-in-HF	-1853.102533	-1853.052473	-1853.024501
DMRG-in-B3LYP	-1856.159181	-1856.088551	-1856.116160
DMRG-in-PBE0	-1855.606274	-1855.536965	-1855.559453
DMRG-in-HF	-1853.146749	-1853.072713	-1853.093745

^{a}CCSD performed in Orca preceeded by HF with DIIS convergence acceleration.
^{b}CCSD performed in Q-Chem preceeded by HF with GDM convergence acceleration.

Natural orbitals of $[Fe(CN)_5(NO)]^{2-}$ complex

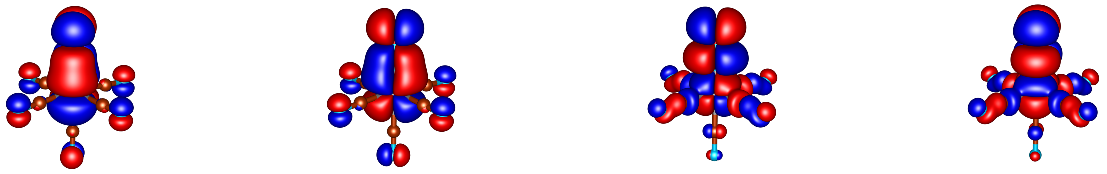

(1) $n_{\mathrm{occup}} = 1.8235$ (2) $n_{\mathrm{occup}} = 1.8231$ (3) $n_{\mathrm{occup}} = 0.1765$ (4) $n_{\mathrm{occup}} = 0.1765$

Figure S2: Fe-NO complex, standard, CASSCF(4, 4)

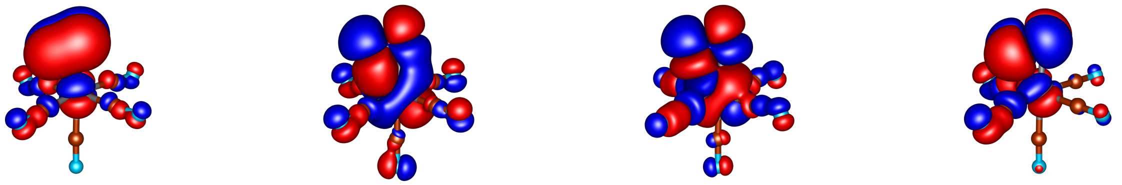

(1) $n_{\mathrm{occup}} = 1.9024$ (2) $n_{\mathrm{occup}} = 1.6449$ (3) $n_{\mathrm{occup}} = 0.3550$ (4) $n_{\mathrm{occup}} = 0.0977$

Figure S3: Fe-NO complex, flat, CASSCF(4, 4)

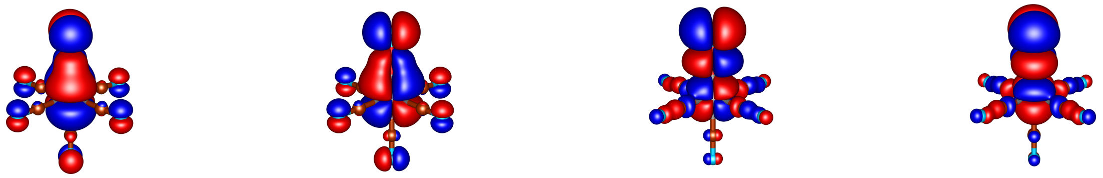

(1) $n_{\mathrm{occup}} = 1.6793$ (2) $n_{\mathrm{occup}} = 1.6679$ (3) $n_{\mathrm{occup}} = 0.3321$ (4) $n_{\mathrm{occup}} = 0.3207$

Figure S4: Fe-NO complex, reverse, CASSCF(4, 4)

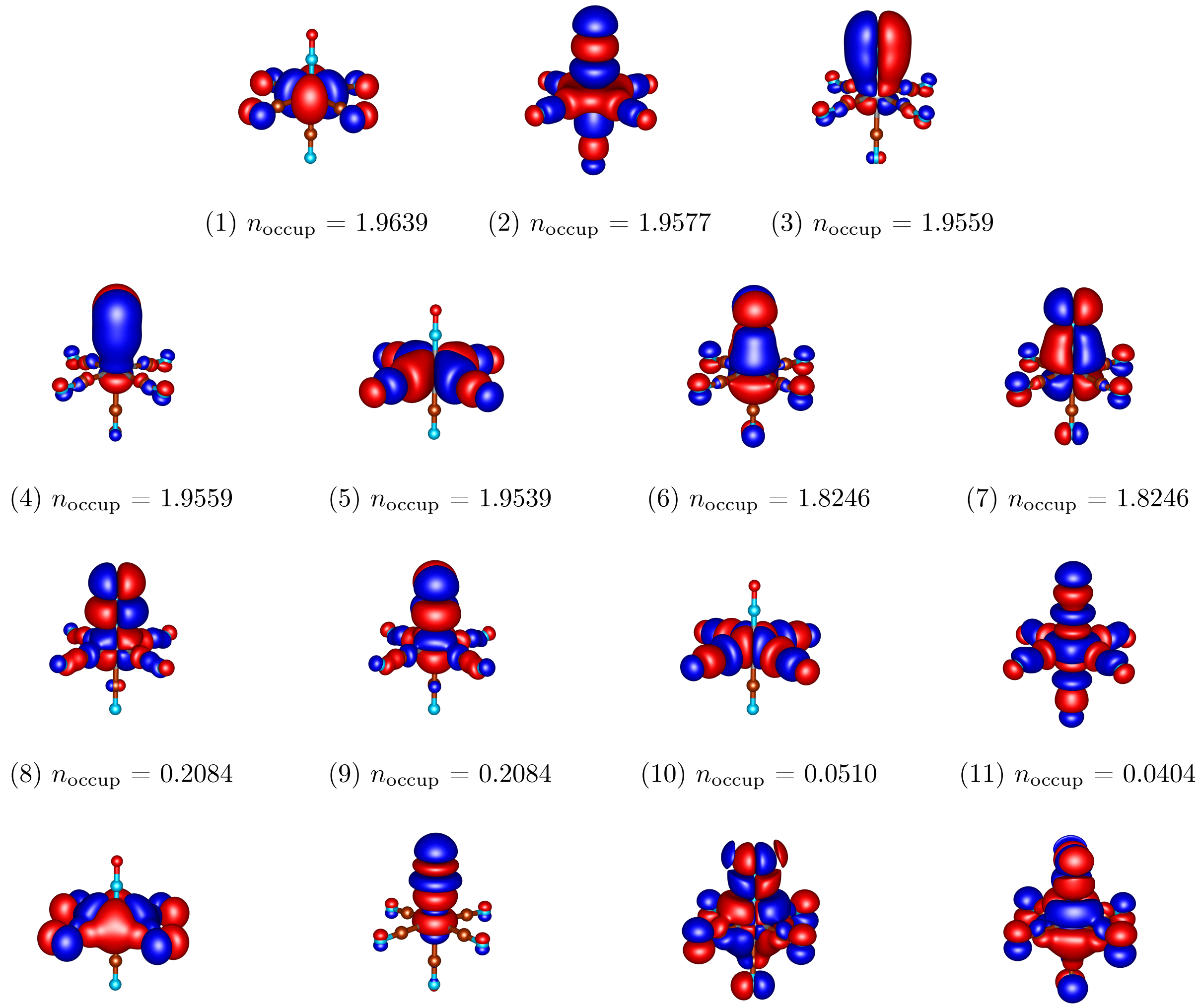

(1) $n_{\mathrm{occup}} = 1.9639$ (2) $n_{\mathrm{occup}} = 1.9577$ (3) $n_{\mathrm{occup}} = 1.9559$

(4) $n_{\mathrm{occup}} = 1.9559$ (5) $n_{\mathrm{occup}} = 1.9539$ (6) $n_{\mathrm{occup}} = 1.8246$ (7) $n_{\mathrm{occup}} = 1.8246$

(8) $n_{\mathrm{occup}} = 0.2084$ (9) $n_{\mathrm{occup}} = 0.2084$ (10) $n_{\mathrm{occup}} = 0.0510$ (11) $n_{\mathrm{occup}} = 0.0404$

(12) $n_{\mathrm{occup}} = 0.0204$ (13) $n_{\mathrm{occup}} = 0.0156$ (14) $n_{\mathrm{occup}} = 0.0095$ (15) $n_{\mathrm{occup}} = 0.0095$

Figure S5: Fe-NO complex, standard, CASSCF(14, 15)

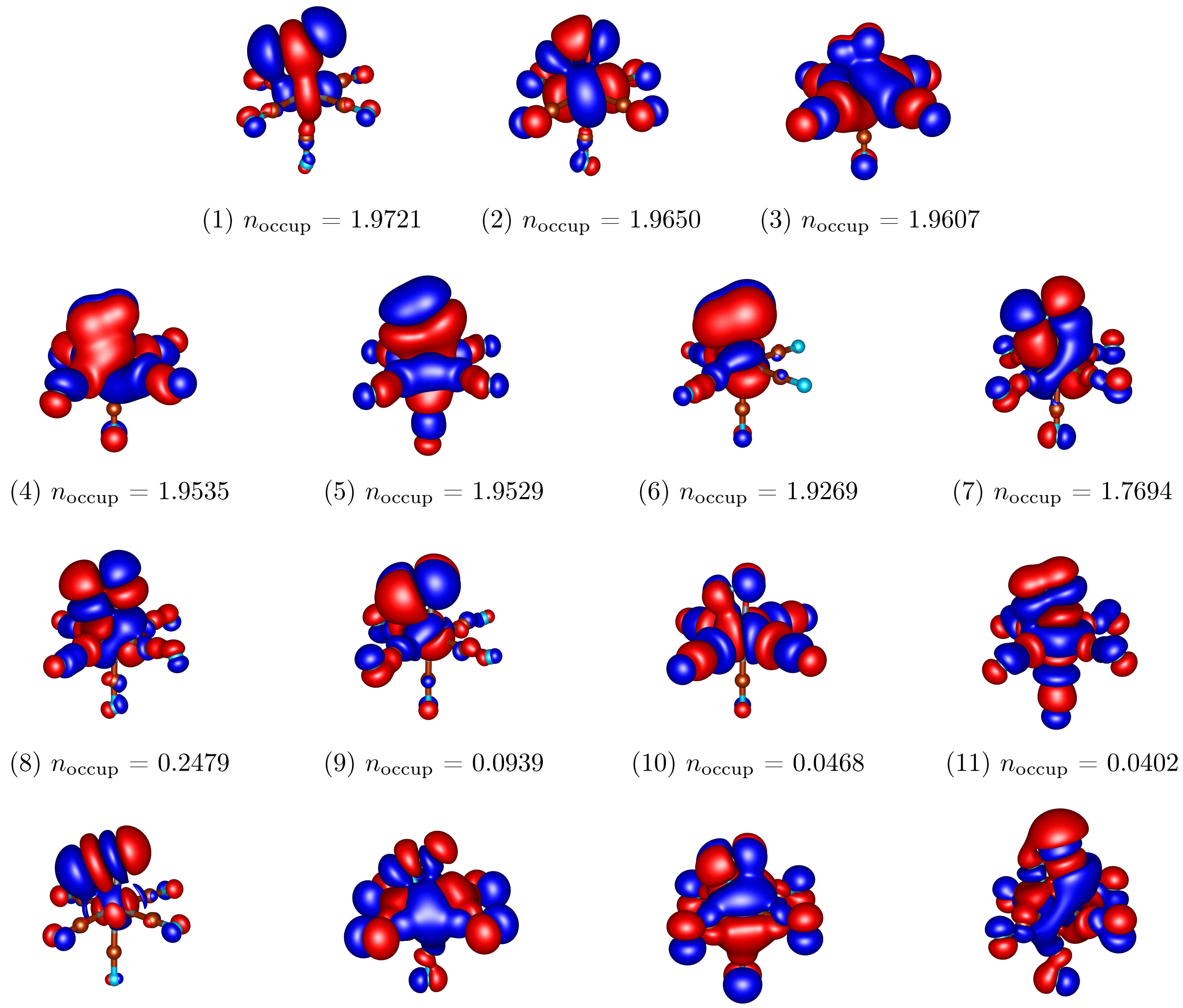

(1) $n_{\mathrm{occup}} = 1.9721$ (2) $n_{\mathrm{occup}} = 1.9650$ (3) $n_{\mathrm{occup}} = 1.9607$

(4) $n_{\mathrm{occup}} = 1.9535$ (5) $n_{\mathrm{occup}} = 1.9529$ (6) $n_{\mathrm{occup}} = 1.9269$ (7) $n_{\mathrm{occup}} = 1.7694$

(8) $n_{\mathrm{occup}} = 0.2479$ (9) $n_{\mathrm{occup}} = 0.0939$ (10) $n_{\mathrm{occup}} = 0.0468$ (11) $n_{\mathrm{occup}} = 0.0402$

(12) $n_{\mathrm{occup}} = 0.0295$ (13) $n_{\mathrm{occup}} = 0.0192$ (14) $n_{\mathrm{occup}} = 0.0140$ (15) $n_{\mathrm{occup}} = 0.078$

Figure S6: Fe-NO complex, flat, CASSCF(14, 15)

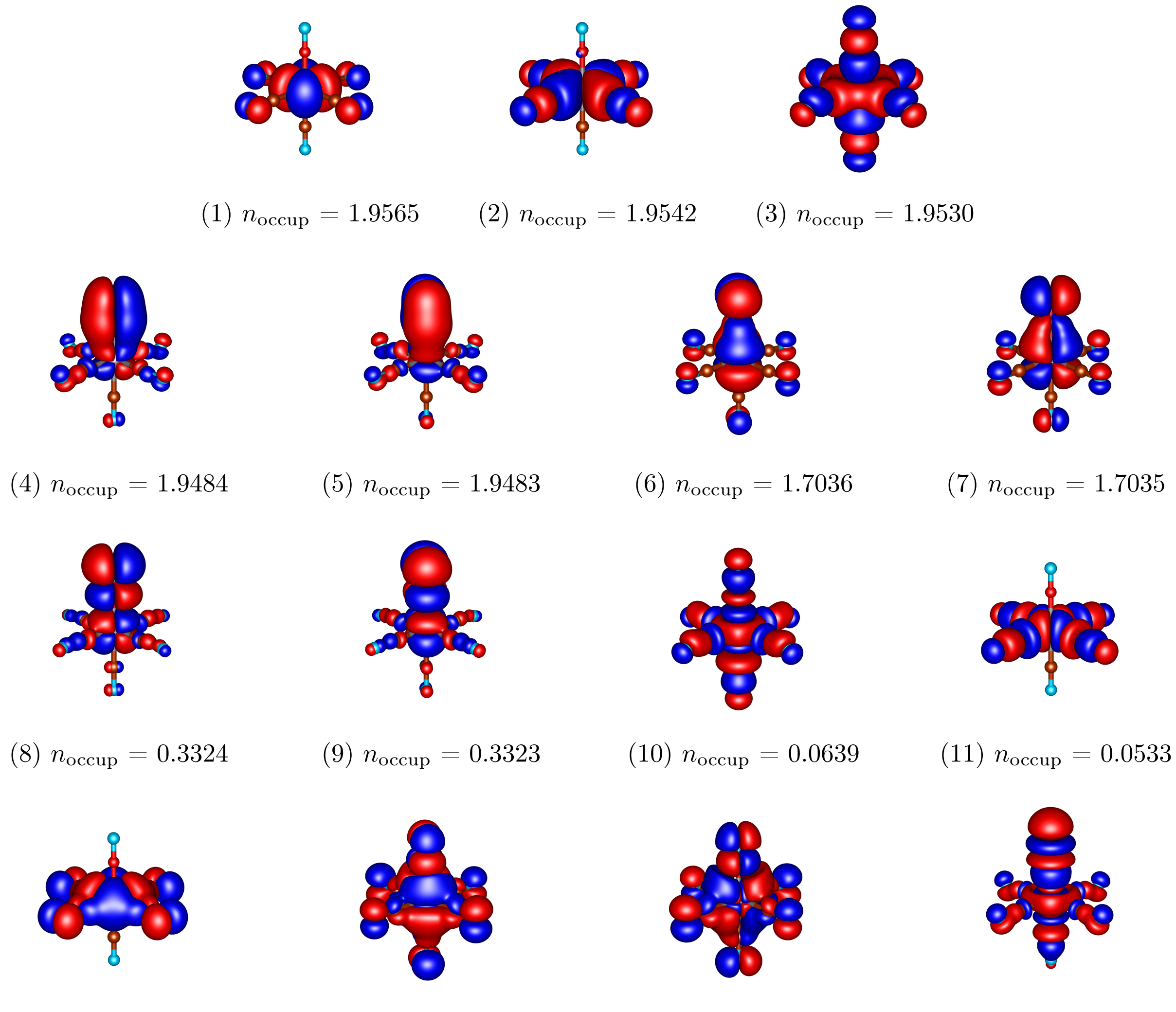

(1) $n_{\text{occup}} = 1.9565$ (2) $n_{\text{occup}} = 1.9542$ (3) $n_{\text{occup}} = 1.9530$

(4) $n_{\text{occup}} = 1.9484$ (5) $n_{\text{occup}} = 1.9483$ (6) $n_{\text{occup}} = 1.7036$ (7) $n_{\text{occup}} = 1.7035$

(8) $n_{\text{occup}} = 0.3324$ (9) $n_{\text{occup}} = 0.3323$ (10) $n_{\text{occup}} = 0.0639$ (11) $n_{\text{occup}} = 0.0533$

(12) $n_{\text{occup}} = 0.0223$ (13) $n_{\text{occup}} = 0.0131$ (14) $n_{\text{occup}} = 0.0131$ (15) $n_{\text{occup}} = 0.0020$

Figure S7: Fe-NO complex, reverse, CASSCF(14, 15)

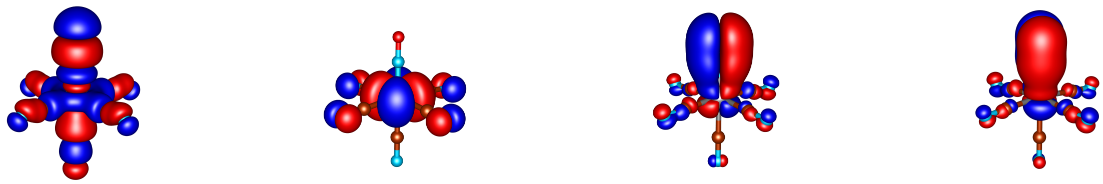

(1) $n_{\text{occup}} = 1.9760$ (2) $n_{\text{occup}} = 1.9624$ (3) $n_{\text{occup}} = 1.9573$ (4) $n_{\text{occup}} = 1.9573$

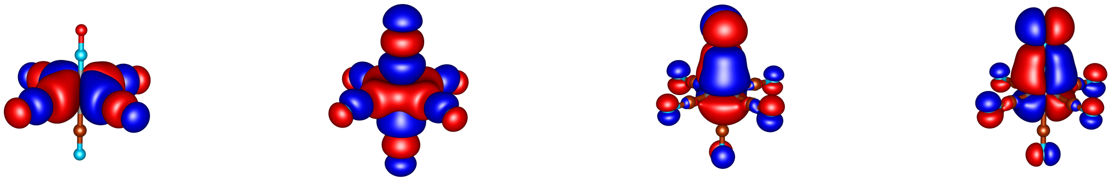

(5) $n_{\text{occup}} = 1.9517$ (6) $n_{\text{occup}} = 1.9502$ (7) $n_{\text{occup}} = 1.8244$ (8) $n_{\text{occup}} = 1.8244$

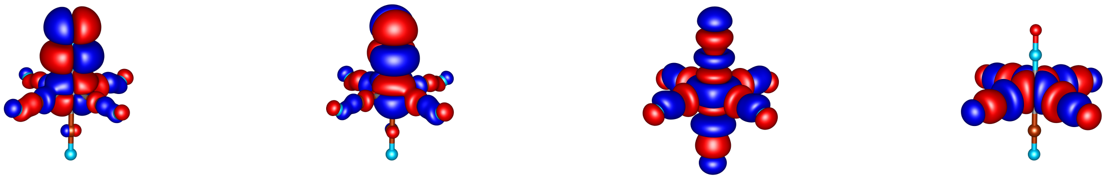

(9) $n_{\text{occup}} = 0.2060$ (10) $n_{\text{occup}} = 0.2060$ (11) $n_{\text{occup}} = 0.0610$ (12) $n_{\text{occup}} = 0.0537$

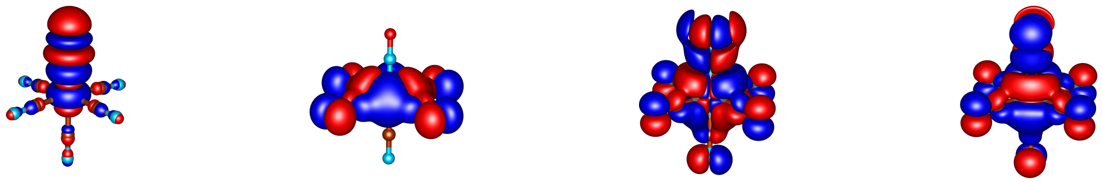

(13) $n_{\text{occup}} = 0.0253$ (14) $n_{\text{occup}} = 0.0227$ (15) $n_{\text{occup}} = 0.0109$ (16) $n_{\text{occup}} = 0.0109$

Figure S8: Fe-NO complex, standard, DMRG-SCF(16, 16)

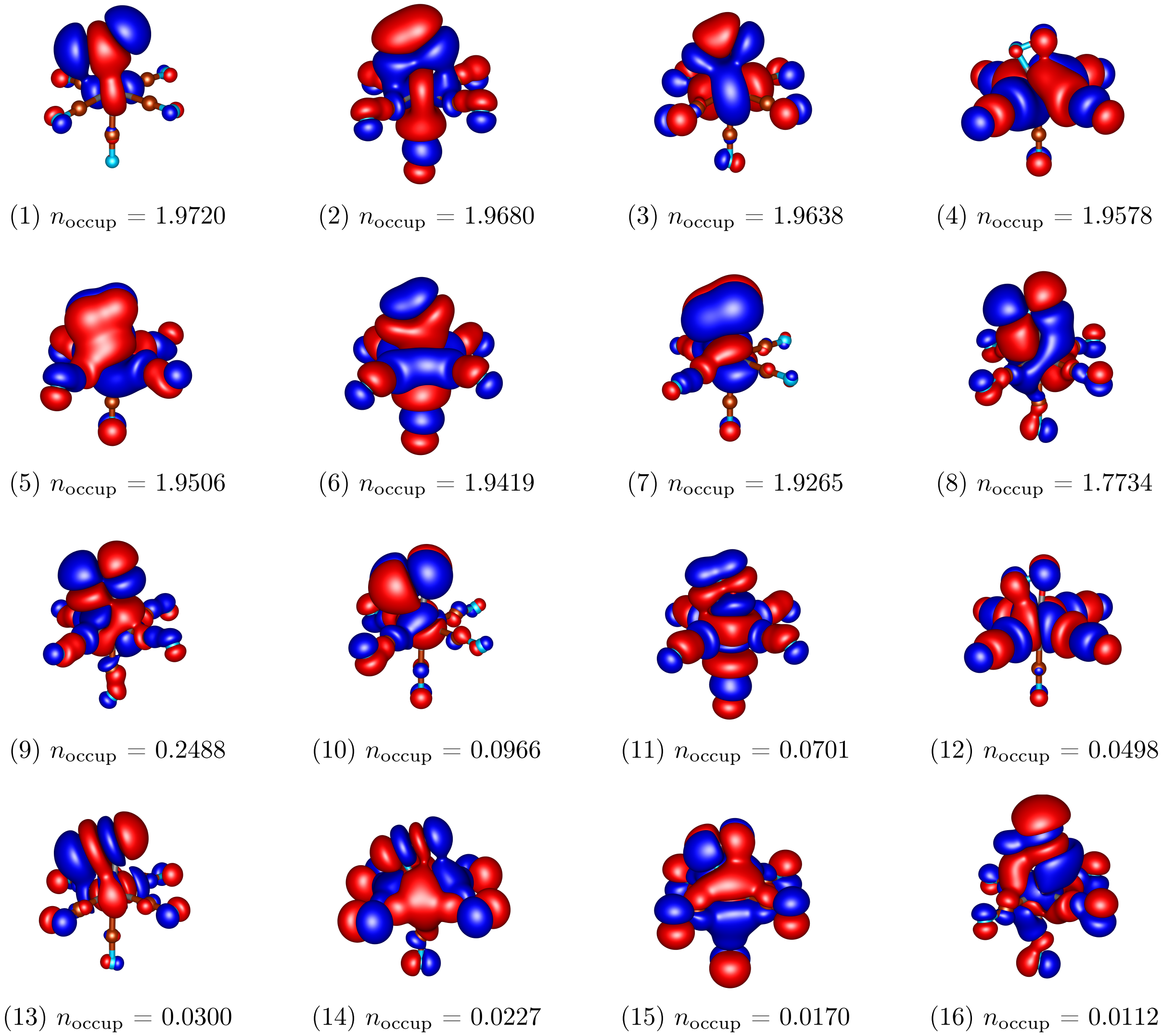

Figure S9: Fe-NO complex, flat, DMRG-SCF(16, 16)

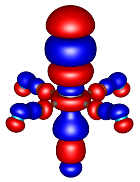
(1) $n_{\text{occup}} = 1.9768$

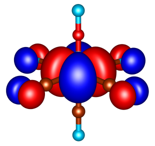
(2) $n_{\text{occup}} = 1.9572$

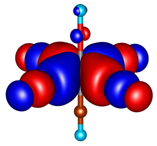
(3) $n_{\text{occup}} = 1.9538$

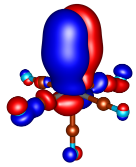
(4) $n_{\text{occup}} = 1.9518$

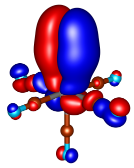
(5) $n_{\text{occup}} = 1.9518$

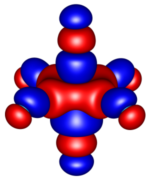
(6) $n_{\text{occup}} = 1.9488$

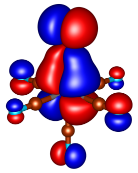
(7) $n_{\text{occup}} = 1.7169$

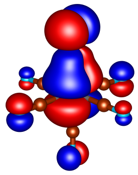
(8) $n_{\text{occup}} = 1.7167$

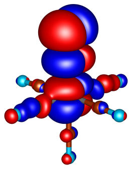
(9) $n_{\text{occup}} = 0.3151$

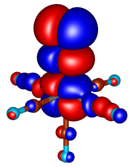
(10) $n_{\text{occup}} = 0.3149$

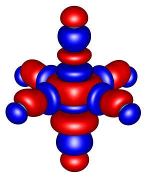
(11) $n_{\text{occup}} = 0.0682$

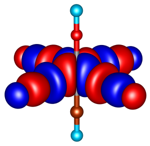
(12) $n_{\text{occup}} = 0.0540$

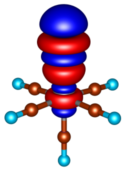
(13) $n_{\text{occup}} = 0.0235$

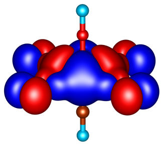
(14) $n_{\text{occup}} = 0.0230$

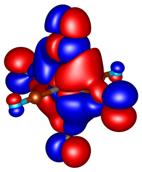
(15) $n_{\text{occup}} = 0.0138$

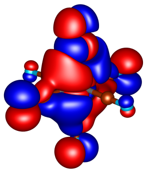
(16) $n_{\text{occup}} = 0.0138$

Figure S10: Fe-NO complex, reverse, DMRG-SCF(16, 16)
